# Laser Welding of Transmitting High-Performance Engineering Thermoplastics

**DOI:** 10.3390/polym12020402

**Published:** 2020-02-10

**Authors:** Fábio A.O. Fernandes, António B. Pereira, Bernardo Guimarães, Tiago Almeida

**Affiliations:** TEMA—Centre for Mechanical Technology and Automation, Department of Mechanical Engineering, University of Aveiro, Campus de Santiago, 3810-193 Aveiro, Portugal

**Keywords:** pulsed Nd:YAG laser welding, high-performance engineering thermoplastic, ERTALON 6 SA, polyamide 6, mechanical testing, numerical model

## Abstract

Laser processing is a rapidly growing key technology driven by several advantages such as cost and performance. Laser welding presents numerous advantages in comparison with other welding technologies, providing high reliability and cost-effective solutions. Significant interest in this technology, combined with the increasing demand for high-strength lightweight structures has led to an increasing interest in joining high-performance engineering thermoplastics by employing laser technologies. Laser transmission welding is the base method usually employed to successfully join two polymers, a transmitting one through which the laser penetrates, and another one responsible for absorbing the laser radiation, resulting in heat and melting of the two components. In this work, the weldability of solely transmitting high-performance engineering thermoplastic is analyzed. ERTALON^®^ 6 SA, in its white version, is welded by a pulsed Nd:YAG laser. Tensile tests were performed in order to evaluate the quality of each joint by assessing its strength. A numerical model of the joint is also developed to support the theoretical approaches employed to justify the experimental observations.

## 1. Introduction

Currently, laser processing is a key technology, addressing some major manufacturing challenges. Laser welding has become a very competitive technology [1]. This relatively modern and innovative technology presents several advantages, such as no contact, high flexibility, and easy handling. Automated laser welding systems are highly reliable, providing high quality, productivity, and cost-effective solutions.

The extensive list of industrial applications for laser welding of polymers has been growing substantially. The automotive and aerospace industries, as well as the medical and electronic sectors, are some examples. This significant increasing demand is justified by the common requirements of high-quality, durable, reliable welds, which are essential in high-performance applications, especially when the welds are exposed to a wide span of environmental and load conditions [2,3], as well as by the low cost and ease of manufacture of high-strength polymeric materials and their lightweight composites [4,5,6,7,8].

Although laser welding was initially employed to join metals, its application was extended to polymers, thanks to the increasing use of solid-state lasers [1]. In particular, a solid-state laser such as the Nd:YAG laser emits a short-wavelength (1060 nm) infrared beam, making it ideal for high-speed and robotic welding [9,10]. In laser transmission welding, the laser wavelength is one of the most important parameters since the optical properties of polymers are wavelength dependent [11,12].

Laser welding of thermoplastics is a relatively recent and cost-effective joining technique, with many advantages over other joining methods, e.g. mechanical fastening and adhesive bonding. It makes it possible to manufacture low-weight complex structures and eases integration with other manufacturing processes, reducing manufacturing times [2]. Additionally, it is even used in particular applications such as the encapsulation of high-performance polymers in medical implants [13]. The concentrated heating and absence of mechanical stresses make this technology well-suited for sensitive applications.

The method typically used to join thermoplastics by a laser is known as laser transmission welding. It is based on the melting caused by the heating associated to the radiation absorbed by the base material [14]. In other words, laser welding of thermoplastics involves a transmitting material through which the laser penetrates and another one responsible for absorbing the laser radiation to generate heat, melting both components.

The two parts must have different transmissive properties in order for the laser beam to be transmitted through one of them (transmitting part) and then absorbed by the other (absorptive part). In addition, full contact is necessary to weld both parts, usually pressed against each other. Pressure is needed to guarantee full contact between samples and improve heat conduction. The contact between both parts guarantees that the transmissive one receives heat by conduction [14,15]. Additionally, in order to achieve a successful weld, both polymers need to be compatible and have similar properties, especially the melting temperature [16]. On the other hand, thermoplastics have significantly lower melting points and thermal conductivity than metals, making them highly sensitive to the intensity of laser beams.

In the literature, there are several experimental studies on laser welding of thermoplastics, sometimes combined with modeling to predict and optimize the process. For example, this was the approach used by Van de Ven and Erdman [17] for laser welding of polyvinyl chloride (PVC) parts. Later, Acherjee et al. [18] investigated the effects of process parameters, such as laser power, welding speed, laser beam diameter, and clamp pressure on the lap-shear strength and the weld bead width for the laser transmission welding of PVC. Hopmann and Weber [19] presented new advances for the laser transmission welding of dissimilar thermoplastics.

Additionally, laser welding has been used to successfully join fiber-reinforced thermoplastic composites [20,21,22,23,24]. More recently, the influence of laser welding process parameters on the weld bead quality of thermoplastic composites with high moisture content has also been studied [25]. Moisture effects on the properties of laser welded polyamide were investigated [26]. In another study, the optical material properties necessary to assess the weldability of scattering materials were determined for some white polymers, including for polyamide 6 (PA 6) [27].

One of the limitations of laser transmission welding is the need of an absorptive part/material, usually of dark color. One particular challenge is to successfully weld mainly transmitting parts, usually of light colors or even transparent, without employing an absorptive one. The degree of complexity in laser transmission welding is much higher when joining two transmitting components, such as two transparent (to laser) parts, being even greater for white parts [12].

This work contributes to transmission welding of white polymers, which are considered the most difficult to weld. Therefore, in this work, the weldability of white high-performance engineering thermoplastic is analyzed. In a previous work, the white and black variants of Ertalon 6 SA, a form of PA 6, were successfully weld [28]. In the current study, the transmitting version of Ertalon 6 SA, the natural (white) version, is welded by a pulsed Nd:YAG laser. Tensile tests were performed in order to evaluate the quality of the welds performed by assessing the joint strength. A numerical model of the joint is also developed to support the theoretical approaches employed to justify the experimental observations.

## 2. Materials and Methods

The main aim of this work was to determine the weldability of the transmitting version of an engineering thermoplastic, the white version of ERTALON 6 SA (natural). Polyamide is a semi-crystalline thermoplastic that presents an interesting set of properties for several engineering applications [29,30] e.g., it is easy to process, has high mechanical strength, stiffness, hardness and toughness, good fatigue resistance, high mechanical damping ability, excellent wear resistance, and good resistance to high energy radiation (gamma- and X-rays). Additionally, polyamide can absorb and reflect a considerable portion of laser energy without additives, presenting good optical properties and very good welding characteristics. This material had already been used by the authors in a previous work, where the influence of pulsed laser on the mechanical characteristics of welded PA6 joints was studied [28]. Laser processing of PA has been also employed for additive manufacturing. The influence of laser-sintering of PA on the mechanical and geometrical parameters was assessed by Stoia et al. [7] and previously explored by Goodridge et al. [31].

The Ertalon 6 SA was supplied by Quadrant Plastics in its white version. Table 1 and Table 2 present indicative values of some mechanical and thermal properties accordingly to the manufacturer [32]. It is also worth to refer that the yield stress ranges from 45 to 80 MPa, depending on the test conditions (dry material or 23 °C/50% RH, respectively). Table 3 presents some optical properties for standard polyamide 6 in its natural version, which are relevant for laser transmission welding.

### 2.1. Laser Welding

The main goal is to determine the weldability of Ertalon 6 SA white version, and thus an optimal set of parameters that makes it possible to join two transmitting components. Therefore, an optimization of the welding parameters is carried in order to successfully weld this polymer, aiming for similar mechanical properties of the base material.

The samples were welded in the Sisma SWA300 Nd:YAG laser machine, using a fundamental wavelength of 1064 nm. It has an average maximum power of 300 W and a peak pulse power of 12 kW. It has already been used to carry out other welding studies, from high-strength steels to dissimilar alloys [33,34], as well as to weld the two versions (white and black) of the material herein studied [28]. This machine makes it possible to set parameters such as the laser power, pulse duration, and frequency, percentage of overlapping between beams, and also their diameter.

Based on the literature and on the authors experience, the first set parameters was defined as described in Table 4. The other parameters were chosen based on an iterative process, based on the immediate observations after welding with the previous set. Special attention was given to the pulse type, its width (duration), and how it influences the energy. The focus on the pulse width is related with its direct influence on the weld strength. On the other hand, the pulse shape influence is also carefully analyzed since it is usually a disregarded parameter.

#### 2.1.1. Joint Configuration

The material was obtained in the form of rods with a diameter of 30 mm. These were cut into 1 mm thick discs, which will be used as samples. These were polished in order to achieve smooth and uniform surfaces.

**Figure 1:**
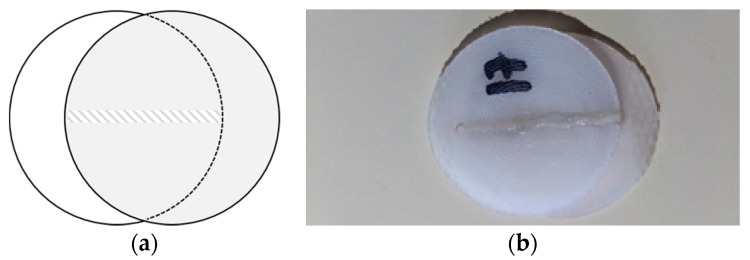
Longitudinal overlap joint configuration: (**a**) Illustration; (**b**) Welded sample.

The welding configuration adopted was an overlap configuration, which is the typical joint configuration for the transmission welding of polymers. However, as already referred, usually one of the parts is transparent or highly transmissible and the other component is made of an absorbing material. The challenge in this work is to successfully join two parts made of the same transmissible polymer. A schematic representation of the lap joint configuration is illustrated in Figure 1. The longitudinal joint aligned with the loading direction is able to withstand higher loads than a transversal one.

#### 2.1.2. Clamp System

**Figure 2:**
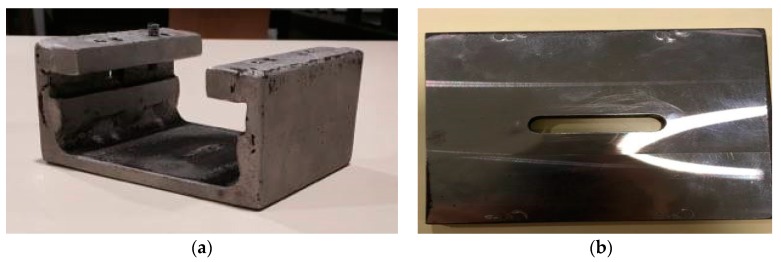
Clamp system: (**a**) main structure; (**b**) slotted plate.

A clamp system from previous works was adapted. In order to perform the lap joints, this suffered some minor modifications to guarantee a proper contact between the parts to weld, by applying pressure on both. Figure 2 shows the main support structure and the slotted plate that will press the components to weld against each other thanks to four M6 screws. The slotted plate presses the components to weld thanks to the action of these screws. By tightening them, these press the slotted plate down, which presses the components to weld against the base of the main structure.

### 2.2. Mechanical Testing

In order to check the quality of the welds, and thus its mechanical strength, tensile tests were performed in a Shimadzu AGX 10 kN universal testing machine at a speed of 1 mm/min. The tensile tests do not follow any particular standard, mainly due to the size limitations of the samples, imposed by the original size of the supplied rods. For comparison, the base material was also tested, and the results compared to the information provided by the manufacturer. The results presented below show that the size limitation did not affect the results, obtaining realistic tensile strength values.

## 3. Results

### 3.1. Mechanical Testing

**Figure 3:**
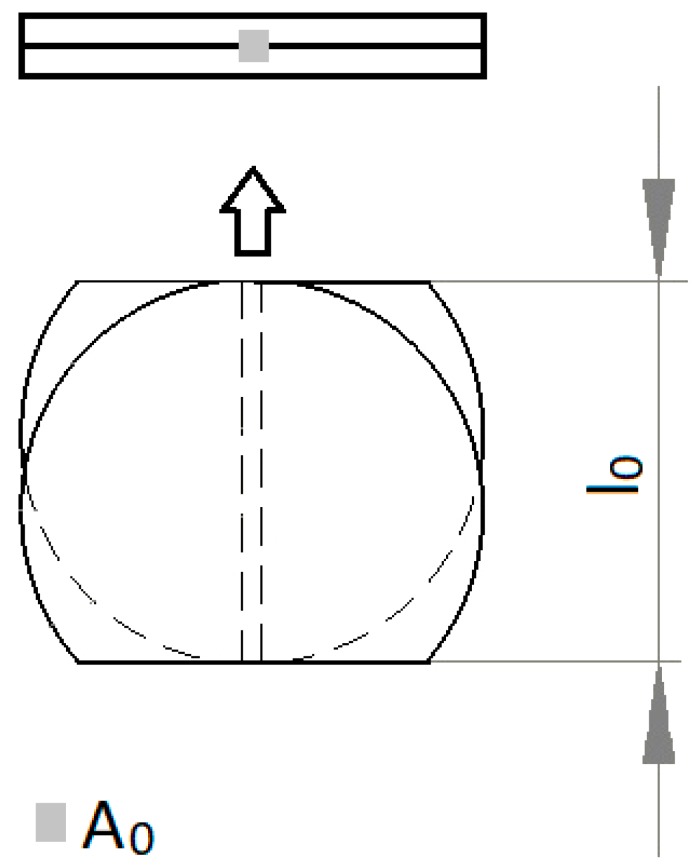
Drawing illustrating the specimen original dimensions and cross-section.

Tensile tests were performed in order to assess the quality of the joints. Three specimens were welded for each set of parameters prior to being subjected to tensile loading. The most representative one is selected for plotting. The results here presented are in the form of engineering stress, σ, and engineering strain, ε. The latter considers the elongation of the gauge length of the specimen, *l*−*l*_0_, and the original gauge length, *l*_0_. The engineering stress is obtained by dividing the load measured (*F*) by the original cross-sectional area of the specimen, *A*_0_. The weld joint dimensions were carefully measured by using an optical microscope for accurate measurements. Figure 3 illustrates the specimen for testing depicting its original dimensions and cross-section.

*σ* = *F*/*A*_0_, (1)

*ε* = (*l−l*_0_)/*l*_0_, (2)

#### 3.1.1. Base Material

**Figure 4:**
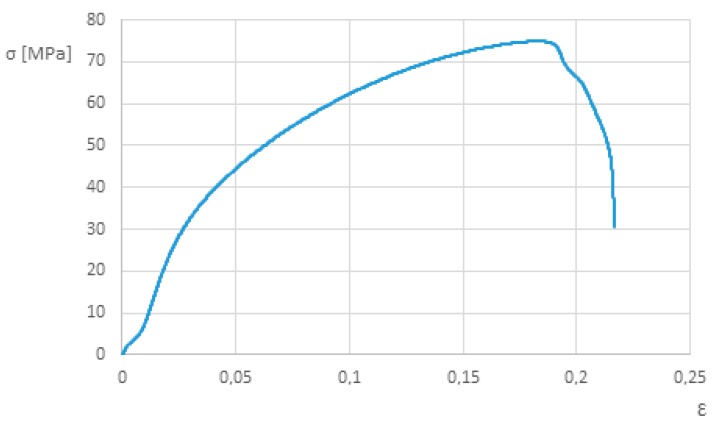
Tensile stress-strain curve of Ertalon 6 SA white.

The specimens failed within the gauge length (Figure 4). The average tensile strength was 74.93 MPa, which is similar to the value obtained in [28] and the value reported by the manufacturer [32]. The yield stress also lies within the ranges (45–80 MPa) reported by the manufacturer. Moreover, the present values agree well with those available in the literature for similar materials [35].

#### 3.1.2. Joints

**Figure 5:**
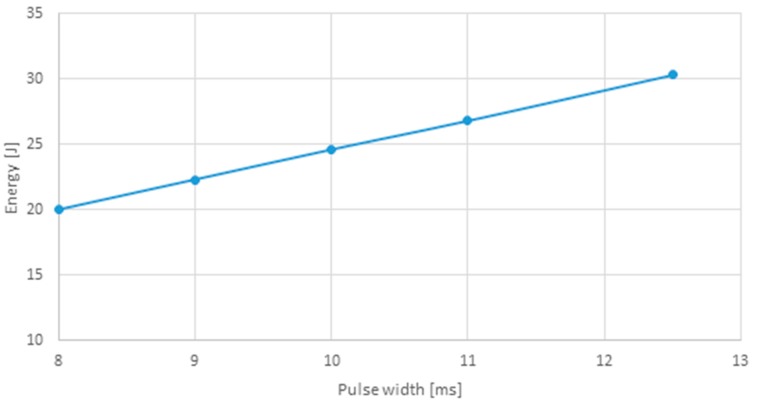
Energy relation with square pulse width (sets 1–5).

**Figure 6:**
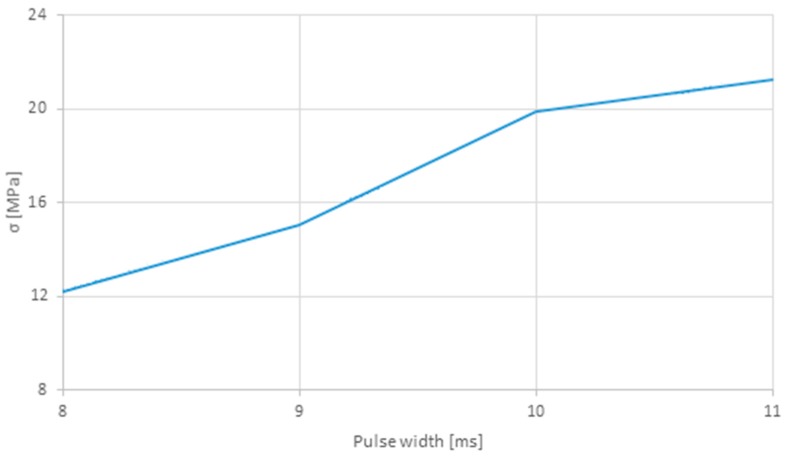
Tensile strength obtained for different square pulse widths (sets 1–5).

Table 5 lists the tensile strength and corresponding maximum force, as well as the weld bead dimensions (length × width) obtained for each set of parameters. Figure 5 plots the energy for each square pulse (sets 1–5) and its width, while Figure 6 presents the tensile strength relation with the pulse duration for the same sets. This comparison was performed since these sets have the same pulse shape, varying only its duration.

By increasing the pulse width, the amount of energy is also increased, leading to higher forces and thus to joints with higher tensile strengths. Therefore, pulse width is a crucial parameter, with a significant influence on the strength and quality of the weld joint.

In the next sets, 6 to 10, the shape of the pulse was changed in order to determine its influence and how it affects the energy and most importantly, the quality of the joints. The type of pulse that resulted in higher energies was the one employed in set 8. On the opposite, the one that resulted in the lower amount of energy was set 10. The fact the strength of sets 6–7 is lower than 8–10 for similar energy levels is probably related with the pulse type. In other words, how the energy is given to the material since the welding parameters are almost the same, with similar welding energies, the pulse type being the main difference.

**Figure 7:**
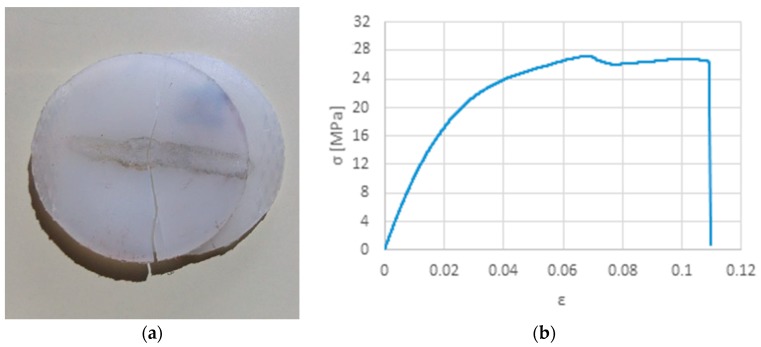
Results obtained with set 9: (**a**) Sample after testing exhibiting a fracture perpendicular to the joint; (**b**) Tensile stress-strain curve.

**Figure 8:**
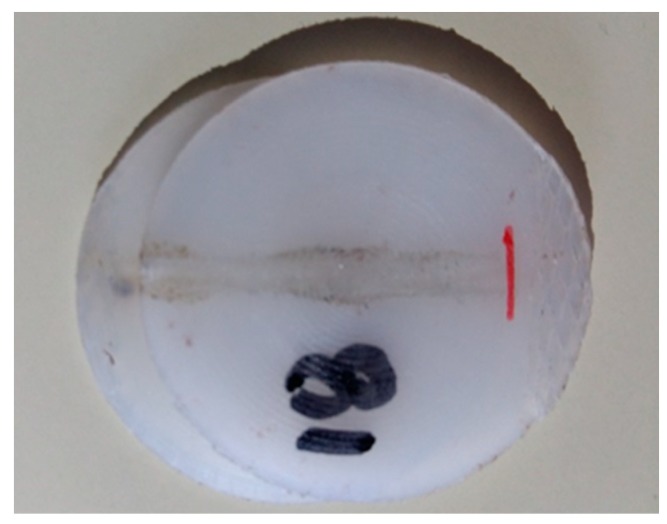
Sample after testing exhibiting interfacial failure (set 10).

Samples welded with set 9, oppositely to some of the previous samples, did not fail in weld seam but rather perpendicularly, showing a weld with superior performance than the formers. However, the tensile strength is still much inferior than the base material, which might reflect a pre-existing micro-fissure that propagated during tensile loading (Figure 7a). Figure 1b shows the same sample prior testing. Figure 8 shows the common mechanism of failure obtained for the other samples, interfacial failure by breaking in the joint.

In the next step, sets 11–12 were carried out based on the previous ones that presented the best results, and by increasing the peak power, the pulse width, and thus the amount of energy. Although the maximum force was lower than what was obtained for samples welded with sets 5–10, samples welded accordingly with sets 11–12 reached higher strengths, considering the shorter joints.

**Figure 9:**
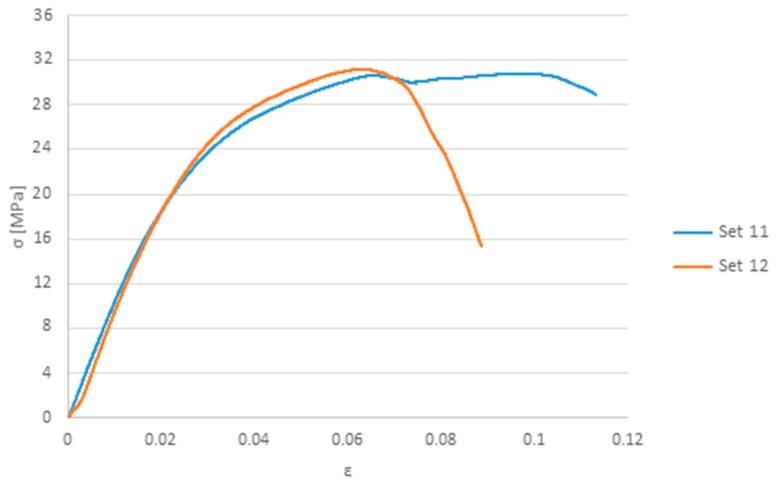
Stress-strain curve obtained for samples welded with sets 11 and 12.

**Figure 10:**
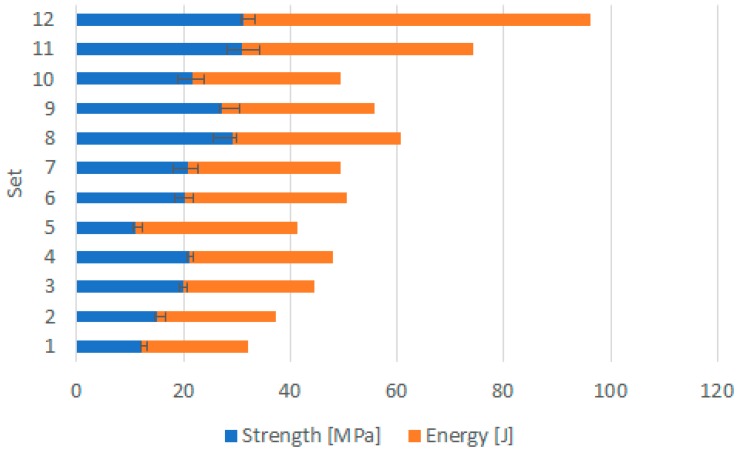
Relationship between energy and strength for each set of welding parameters—error bars indicate the minimum and maximum strength values reached in the tensile tests.

Figure 9 presents a comparison of the stress-strain behavior of samples welded with sets 11 and 12, the ones that exhibited a superior performance. From the results, and focusing on Table 5, it is clear that the welding parameters were set in the right direction, improving the joint strength from set to set. This statement is supported by the relationship between energy and joint strength for each set of welding parameter presented in Figure 10. Although successful welds between the same transmitting material was accomplished, the strength of the weld joints is still far from the base material.

## 4. Numerical Study

A numerical model of the joint is also developed to support the results and the withdrawn conclusions. Although the discs were successfully weld (visually speaking), the strength values obtained were always inferior than base material, rupturing mainly at the joint. Therefore, the aim of modeling the welded samples and simulating the tensile test is to check for possible local stress concentrations in the joint.

### 4.1. Finite Element Modelling

The strategy to model the welded samples consists of a unique part. In other words, a finite element mesh with shared nodes between the discs and the joint. In order to reduce the amount of resources necessary, a simplification was made by modelling just half of the geometry. The symmetry condition does not affect the results and makes it possible to improve the model by increasing the number of elements in the other half.

The joint was modeled with a length of 23 mm. The latter was selected based on the results presented in Table 5. The best joints were obtained with the sets 11 and 12, the ones with shorter joints of 22.1 mm and 22.95 mm, respectively.

A partition-based strategy was also employed to make it possible to model the geometry with hexahedral elements. Two cutting planes, perpendicular to the joint and placed on its endings, were defined, isolating the extremities where the grips are applied. These are also useful to apply the boundary conditions, by fully constraining one end and by applying the displacement on the other. 

**Figure 11:**
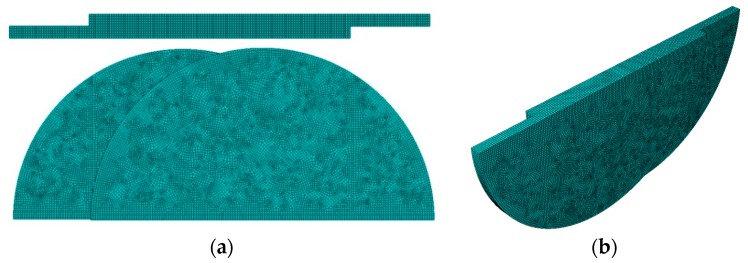
Finite element mesh: (**a**) Top view and joint view showing a well-structured mesh with 0.2 mm hexahedral elements in the joint; (**b**) Isometric view.

Figure 11 presents the numerical model meshed with 108,675 linear hexahedral elements, showing well-shaped 0.2 mm eight-node 3D solid elements on the joint(Abaqus C3D8R). The reduced integrated formulation with hourglass stabilization was employed in order to avoid locking phenomena and other related numerical pathologies [36]. A mesh convergence study was performed in order to eliminate any mesh dependence and also to reduce the computation time necessary.

Although there is only one part, due to the geometry of the model, the discs might interact during the tensile test. In order to avoid these penetrations, a self-contact interaction with surface to surface discretization method was used. The penalty friction formulation was employed with a friction coefficient of 0.2.

#### Constitutive Modeling

The strategy to model the welded samples consists of a sole part with a single set of mechanical properties for the entire model. Although the material properties of the joint were possibly affected by the laser, it presents a small area and the simplification of considering the same material properties for all the model is admissible to determine where the stress concentrations would happen in ideal conditions.

An elastoplastic material model was employed. A mass density of 1.14 g/cm^3^, a Young’s modulus of 3300 MPa and Poisson’s ratio of 0.39 were defined. The strain hardening curve based on the experiments carried out with the base material was used as input to define the plastic behavior. This is the isotropic hardening model, where the yield surface changes size uniformly in all directions such that the yield stress increases (or decreases) in all stress directions as plastic straining occurs [37].

No damage model with element deletion was employed to simulate fracture since the main objective is to determine local stress concentrations. Nevertheless, it is still possible to observe necking formation, which is more than enough considering the objective.

### 4.2. Uniaxial Tensile Test Simulation

**Figure 12:**
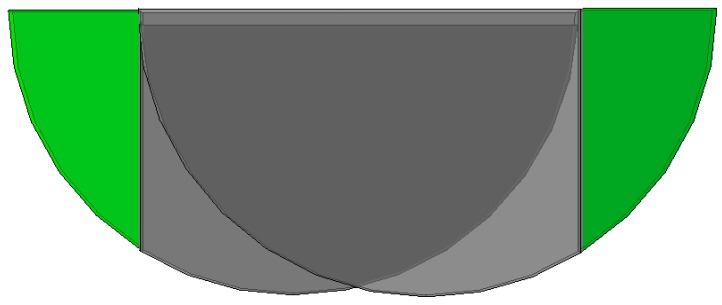
Cell partitioning strategy for definition of the boundary conditions applied on the green regions.

In order to simulate the tensile test, two boundary conditions were assigned on the ends of the model (green regions in Figure 12). One side has all the degrees of freedom restricted. The other end moves only in the axial direction. A displacement is applied, aiming to the level of strain where the maximum force was reached experimentally (Figure 9).

**Figure 13:**
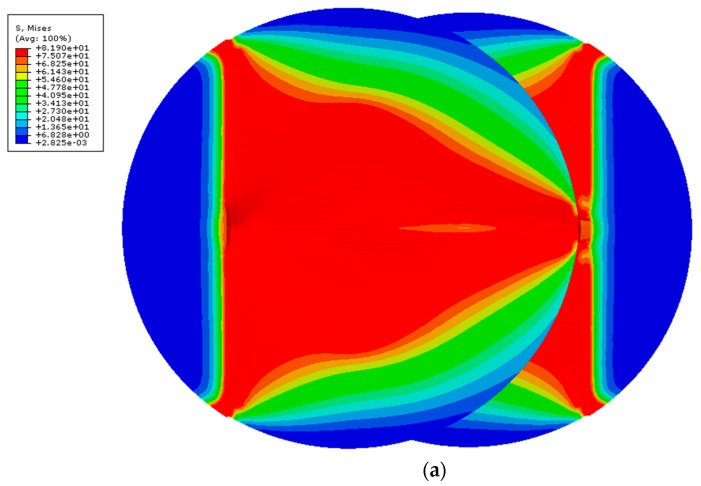

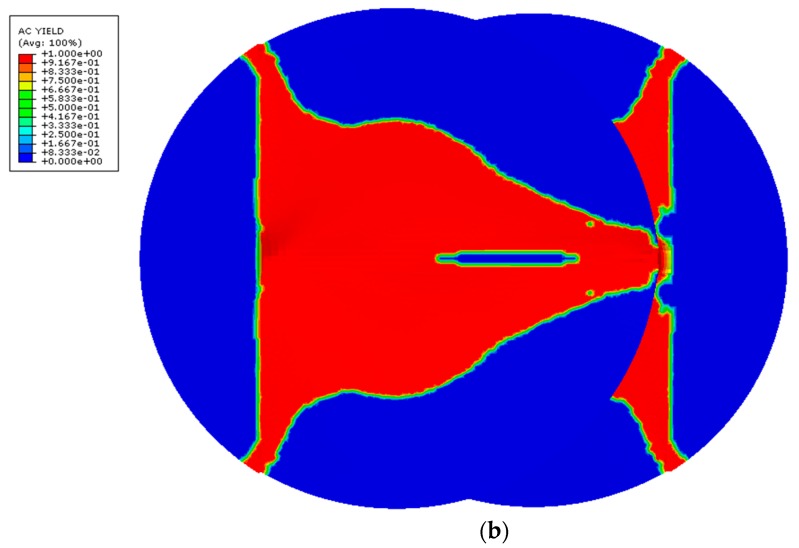
Triangular stress distribution and corresponding yield regions: (**a**) Mapping of von Mises stress; (**b**) Mapping of yielded regions.

The finite element analysis (FEA) of the uniaxial tensile test is presented in Figure 13a, using the implicit integration scheme. By checking the von Mises stress, there is a clear pattern regarding the stress distribution in both discs. Considering there is a symmetry condition, the critical triangular area is probably caused by the action of the grips (triangle base) and the joint (triangle height). Figure 13b) presents the regions where yield was reached, which correlates with the Figure 13a), showing that relatively high stresses are already reached for such a small strain (~0.079).

**Figure 14:**
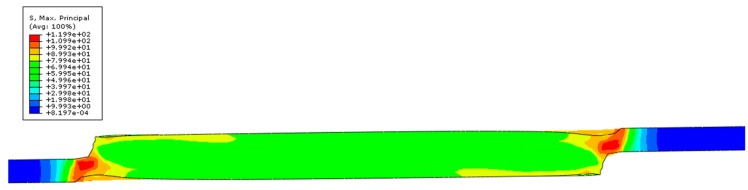
Maximum principal stress—local concentration on the joint end.

Necking behavior was observed in the joint ends as presented in Figure 14. By observing the mapping of the maximum principal stress, there is a non-uniform local concentration in the joint ends. The peak values were found in these regions, where there is a clear stress concentration of almost 120 MPa, which is higher than the tensile strength of the base material. The high stresses reached for such a low strain support the findings of premature failure for relatively low loads, even for this type of joint.

**Figure 15:**
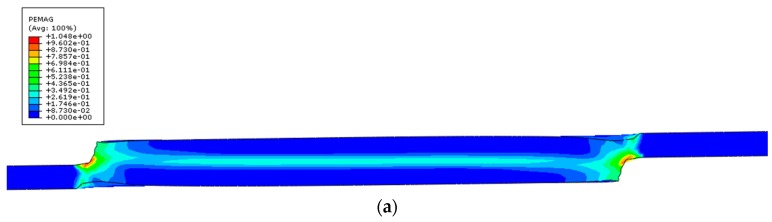

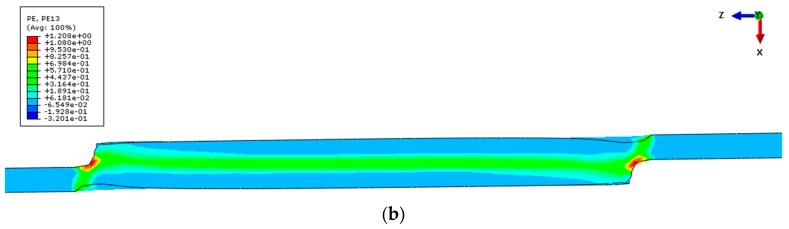
Plastic strain: (**a**) magnitude; (**b**) individual component (X–Z).

Additionally, due to the geometry of the component welded and the nature of this particular mechanical test, the joint is subjected to shear loading. This is evident by the way the joint deforms as depicted in Figure 15, by showing the individual plastic strain component PE13 and the plastic strain magnitude PEMAG.

## 5. Discussion and Conclusions

In this work, the weldability of white Ertalon 6 SA, a form of PA 6, was analyzed. Welding two transmitting components is particularly challenging, especially in the case of white components. The degree of complexity in laser transmission welding of white thermoplastics is significant, even more so than welding two transparent parts [12]. Although in the literature it is possible to find studies that explore the spacing between components to weld [38], in this work, and due to the nature of the material and the joining process, the contact between samples is of outermost importance. Primary tests made it possible to conclude that spacing between samples would be an issue in joining the components, justifying the need for a clamp system to pressure both pieces against each other during the welding process, eliminating gaps and increasing heat conductivity.

A parametric study was carried out by testing several samples welded with different sets of parameters with a pulsed Nd:YAG laser. The incremental evolution of the welding parameters, resulted in a consistent improvement of the quality of the weld joints, with better aesthetics and mainly in the higher strengths. The laser energy density necessary to achieve the best performance was 8.26 × 10^3^ J/cm^2^. However, the results from the tensile tests revealed a joint always weaker than the base material. In a previous work, the white and black variants of Ertalon 6 SA were successfully weld [28]. Nevertheless, the joint strength was also inferior than the tensile strength of the base material.

In future works, higher energy densities should be considered, not just by means of power, but also beam size. Additionally, overlapping should be explored in a future work. These are all parameters, not fully explored in the present work, that influence the melting of the material and thus, the joining process.

A numerical model of the joint was developed to determine possible causes for such behavior. The aim of simulating the tensile test was to check for local stress concentrations near the joint since failure was mainly observed in this region. The results from the simulations revealed necking behavior and non-uniform local stress concentrations in the joint ends. The high stress concentration for low strains support the findings of premature failure for relatively low loads, even for this type of joint that theoretically has a higher tensile strength than a transversal one. Additionally, due to the geometry of the component welded (including the joint configuration), the joint is subjected to shear during tensile loading.

Although the joint strength values are still far from the base material performance, this work contributes to the transmission welding of white polymers, considered the most difficult to weld, by establishing an excellent background for future studies, by identifying the main mechanisms of failure and the way the welding parameters need to be optimized to improve the mechanical strength of the joint. Additionally, to the best of our knowledge, this is the first time Ertalon 6 SA white components were welded, most notably with a Nd:YAG pulsed laser.

## Figures and Tables

**Table 1:** Density and thermal properties of Ertalon^®^ 6 SA [32].

Density [g/cm^3^]	Melting Temp. [°C]	Thermal Conductivity [W/(K.m)] ^1^	Coef. of Linear Thermal Expansion [m/(m.K)] ^2^
1.14	220	0.28	90–105 × 10^−6^

^1^ @ 23 °C; ^2^ average value between 23–60 °C and 23–100 °C.

**Table 2:** Mechanical properties of Ertalon^®^ 6 SA under tensile loading (ISO 527-1/-2) at 23 °C [32].

Tensile Strength [MPa]	Yield Strain [%]	Strain at Break [%]	Modulus of Elasticity [MPa]
80	4	> 50 ^1^/100 ^2^	3300 ^1^/1425 ^2^

^1^ Dry material; ^2^ Material in equilibrium with the standard atmosphere 23 °C/50% RH.

**Table 3:** Optical properties of Polyamide 6—natural version [14].

Refractive Index	Heat Capacity (J·K^−1^)	Crystallinity (%)	Peak Absorbance (nm)	Absorbance
1.53	1.5	15–45	1390	0.98

**Table 4:** Welding parameters.

N	Power [%]	Pulse [ms]	Overlapping [%]	Beam Diameter [mm]	Energy [J]	Pulse Type
1	25	8	50	1	20	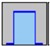
2	25	9	50	1	22.3	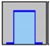
3	25	10	50	1	24.6	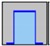
4	25	11	50	1	26.8	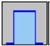
5	25	12.5	50	1	30.3	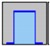
6	25	12.5	50	1	30.3	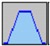
7	25	12.5	50	1	28.5	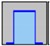
8	25	12.5	50	1	31.4	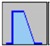
9	25	12.5	50	1	28.7	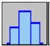
10	25	12.5	50	1	27.9	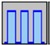
11	30	15	50	1	43.3	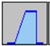
12	40	17.5	50	1	64.9	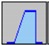

**Table 5:** Results of the tensile tests: tensile strength, maximum force and joint dimensions (length × width) for each set of parameters.

Set	Tensile Strength [MPa]	F_max_ [N]	Joint (Length × Width) [mm]
1	12.18	341.24	28 × 1
2	15.04	391.04	26 × 1
3	19.86	575.85	29 × 1
4	21.24	573.23	27 × 1
5	11.14	307.54	27 × 1
6	20.30	547.89	27 × 1
7	20.80	520.36	25 × 1
8	29.18	729.55	25 × 1
9	27.16	660.14	24.3 × 1
10	21.58	604.39	28 × 1
11	30.84	681.45	22.1 × 1
12	31.19	715.71	22.95 × 1
